# Barriers of Female Breast, Colorectal, and Cervical Cancer Screening Among American Indians—Where to Intervene?

**DOI:** 10.3934/publichealth.2016.4.891

**Published:** 2016-10-31

**Authors:** Yan Lin, Xi Gong, Richard Mousseau

**Affiliations:** 1 Department of Geography & Environmental Studies, University of New Mexico, Albuquerque, New Mexico 87131, USA; 2 Community Health Department, The Great Plains Tribal Chairmen's Health Board, Rapid City, South Dakota 57702, USA

**Keywords:** American Indians, cancer screening, geographic access, barriers

## Abstract

Female breast, colorectal, and cervical cancer are three common cancers among people in the United States. Both their incidence and mortality rates can be dramatically reduced if effective prevention and intervention programs are developed and implemented, because these cancers are preventable through regular screenings. American Indians in the United States especially in the Northern Plains have a disproportionally high burden of these cancers. As a hard-to-reach population group, less attention has been paid to American Indians regarding cancer screening compared with other population groups. This study examined barriers experienced by American Indians residing in South Dakota regarding three cancer sites: female breast, colorectal, and cervical cancer through a community-based survey. A total of 199 participants were recruited and factors significantly associated with cancer screening included knowledge about cancer screening, geographic access to PCPs, encouragement by doctors, as well as socioeconomic barriers. Meanwhile, integrating geographic access, socioeconomic deprivation, and geographic distribution of American Indians, the study identified geographic areas of low access to cancer screening where hard-to-reach populations resided. Results from the study will provide crucial information for the development of targeted intervention programs to increase the acceptability and uptake of cancer screening among American Indians.

## 1. Introduction

Female breast, colorectal, and cervical cancer are three common cancers among people in the United States [1]. Because these cancers are preventable through regular screening, both their incidence and mortality rates can be dramatically reduced if effective prevention and intervention programs are developed and implemented. Screenings can detect and remove precancerous growths, or detect cancer at an early stage when treatment is more successful [1]. For breast cancer, mammography screening is associated with a 15%–20% mortality reduction [2]. At least 80% of cervical cancer incidence and mortality rates can be reduced by regular Pap test [3]. For colorectal cancer, Fecal Occult Blood Test is associated with a 15%–33% reduction in cancer risk; whereas sigmoidoscopy or colonoscopy is associated with about 60%–70% reduction in the risk [4]. Although significant progress has been made in the last few decades, the three cancers remain common among people in the United States. The American Cancer Society estimates that there will be 394,140 newly diagnosed cases (incidence) and 93,760 mortality cases in the United States in 2016 alone for these three cancer sites [1].

American Indians in the United States especially in the Northern Plains have a disproportionally high burden of female breast, cervical, and colorectal cancer [5]. In South Dakota, there will be 1,240 incidences in 2016 alone for these three cancer sites [1]. Incidence and mortality rates for American Indians in South Dakota are higher than those among non-Hispanic whites or the overall population in the United States. Those residing in western South Dakota experienced at least 30% higher incidence and mortality rates than the overall population in the United States [6]. Many of these new cases and deaths could be avoided if these American Indians in underserved communities take routine screening tests. A significant challenge to cancer disparity reduction is to develop screening programs that will help increase cancer screening among these minorities. However, American Indians have been labeled by past researchers “hard-to-reach” because data or program participation are difficult to obtain [7]. Living in isolated or remote reservations with inaccurate addresses, household mobility, high proportion of households without phone or internet access, as well as distrust of outsiders makes American Indians hard-to-reach populations in the United States. Therefore, in order to develop successful screening intervention programs, we must first understand screening barriers among American Indians and identify where these hard-to-reach population live.

Cancer screening among the American Indian population group hasn't attracted as much attention as that among other minority population groups (e.g., African Americans and Hispanics) in the United States. Based on the Centers for Disease Control and Prevention (CDC), the screening rate is 69.4% for breast cancer, 78.7% for cervical cancer, and 49.5% for colorectal cancer among American Indians in the United States. Among African Americans, the screening rate is 73.2% for breast cancer, 85% for cervical cancer, and 55% for colorectal cancer. Among Hispanics, the rate is 69.7% for breast cancer, 78.7% for cervical cancer, and 46.5% for colorectal cancer [8]. There is a lack of nationally based representation of American Indian population regarding cancer screening survey, due to cultural diversity among subgroups of American Indians.

Existing studies that aimed to disentangle cancer screening barriers experienced by American Indian population have found that socioeconomic factors, health literacy, geographic factors, insufficient access to healthcare, and health professionals' encouragement about cancer screening were associated with cancer screening [6],[9]–[14]. However, few studies have been conducted to understand cancer screening barriers and to address strategies about cancer screening intervention programs among American Indians in South Dakota (the Sioux) who experience great burdens in female breast, colorectal, and cervical cancer.

The objective of this study was to examine barriers experienced by American Indians residing in South Dakota regarding female breast, colorectal, and cervical cancer through community-based surveys. Meanwhile, the study aimed to identify geographic areas with low access to cancer screening where hard-to-reach American Indian populations resided based on the survey results. Results from the study will provide crucial information for the development of targeted intervention programs to increase the acceptability and uptake of cancer screening among American Indians.

## 2. Materials and Methods

### 2.1. Study Population

The study population of this research was American Indians over the age of 21 in South Dakota. We used direct recruitment method in the study through Native American Intertribal Festival & Traditional Powwow in Rapid City and Yankton. Powwows are Native American people's way of meeting together, to join in dancing, singing, visiting, renewing old friendships, and making new ones, which attract Native Americans from all over South Dakota. We randomly asked 400 Native American attendees to participate in the study survey and a total of 257 attendees agreed to participate, resulting in a 64% response rate. Native Americans over the age of 21, who currently resided in South Dakota at the time when the survey was conducted, were eligible to be included in the study. The final sample consisted of 199 eligible participants.

### 2.2. Survey Instruments

We designed our survey based on prior instruments [6],[15]. Our survey instruments also included several questions from the Behavioral Risk Factor Surveillance System (BRFSS). The survey in the current study was 13 page long with 70 questions regarding participants' demographics (e.g., age, gender, tribal affiliation, marital status, education, annual household income, employment, and insurance), self-rated health status, health care providers, travel time to health care services, phone, vehicle, and fresh food access, transportation challenges, whether receiving social service support, daily activities, knowledge, attitudes and behaviors about breast, cervical, and colorectal cancer screening, history of cancer screening, cancer screening results, barriers that might influence cancer screening, as well as satisfaction with health care.

Most questions were designed as multiple choice questions which resulted in categorical variables. The education level was determined by the highest level of school completed (elementary, middle school, some high school, graduated high school, some college, graduated from college, and advanced degrees). The annual household income was categorized into less than $10,000, between $10,000 and $24,999, between $25,000 and $34,999, between $35,000 and $49,999, between $50,000 and $74,999, between $75,000 and $99,999, and $100,000 or more. Employment status was dichotomized into currently employed or unemployed. Occupation and place of work were also asked. Travel time (one-way) to health care services was categorized into less than 10 miles, between 10 miles and 30 miles, between 30 miles and 60 miles, between 60 miles and 90 miles, and more than 90 miles. Frequencies and place of routine daily activities such as getting grocery, spiritual activities, eating, leisure, sports, and attending school were asked.

For breast cancer screening, participants were asked whether they had ever heard of or had a mammogram, the length of time since their last screening, place where a mammogram was taken, and reasons why having never had a mammogram. For cervical cancer screening, participants were asked whether they had ever heard of or had a Pap test, the length of time since their last Pap test, place where a Pap test was given, reasons why having never had a Pap test, as well as knowledge about HPV. For colorectal cancer, participants were asked whether they had ever heard of or had a fecal occult or stool blood test, sigmoidoscopy, or colonoscopy, as well as the length of time since their last screening.

Regarding satisfaction with health care, participants were asked about whether they were provided good health care, whether they were treated with dignity and respect by health care providers, whether they were comfortable talking with doctors when they had health problems, whether they trusted doctors, and whether they had been told to get a cancer screening by a doctor or nurse.

The entire survey was designed as a written survey in English and took approximately 20 minutes to complete. All participants were given an information sheet about this survey and asked to sign a consent form before taking the survey. Each participant was given a $10 Walmart gift card after the written survey was completed as an incentive for participation. This study was approved by the Committee for the Protection of Human subjects at South Dakota State University.

### 2.3. Statistical Analyses

The purpose of this study was to examine the association between the uptake of cancer screening and variables collected from the survey, including demographic variables, knowledge, and attitudes about cancer screening, barriers to cancer screening, as well as satisfaction with health care. Three outcome variables were measured in the study: whether a participant had at least one mammogram for breast cancer screening; whether a participant had at least one Pap smear test for cervical cancer screening; and whether a participant had at least one fecal occult blood test, colonoscopy, or sigmoidoscopy for colorectal cancer screening.

We computed descriptive statistics (frequencies) for categorical variables from the survey in this study. Chi-square test was used to examine the association between cancer screening and these variables. Multivariate analysis could not be implemented due to the small sample size as well as the use of categorical variables in the study.

In order to identify geographic areas of low access to cancer screening, we combined spatial and non-spatial factors. Because travel distance to Primary Care Physicians (PCPs) was a significant variable across colorectal cancer, breast, and cervical cancer screening, we measured geographic access to PCPs as an index of spatial accessibility. There are 780 PCPs at 210 practicing sites in South Dakota. We used the shortest travel time to measure geographic access to PCPs in ArcGIS 10.3. We used socioeconomic deprivation as an index of non-spatial accessibility, an important finding from a previous study [16]. We extracted 7 census-tract level variables from the 2010 Census to construct socioeconomic deprivation index, including the percentage of unemployed, percentage of individuals below the poverty level, percentage of households without a vehicle, percentage of households on public assistance, percentage of households with annual income less than $25,000, percentage of individuals with less than high school education, and percentage of individuals with less than college education.

Standardized Z-scores were calculated to identify geographic areas with significantly low geographic access to PCPs (long travel time) and high socioeconomic deprivation. Because the target population in this study was American Indians, standardized Z-score was also calculated for the percentage of American Indian population. An integration of the above three Z-scores was used to identify geographic areas with low access to cancer screening among American Indian population. The integration was conducted through identifying geographic areas with significant Z-scores for all of the three factors: geographic access to PCPs, socioeconomic deprivation, and the percentage of American Indian Population.

## 3. Results

This study consisted of 199 American Indian participants. Table 1 presents the basic characteristics of the survey participants. About half of the participants were 40 years or older. A total of 58.8% of the participants were female, and 41.2% were male. Table 1 also presents tribal affiliations of the participants in this study.

**Table 1.** Characteristics of American Indian population.

Characteristics	No.	Percentage	Characteristics	No.	Percentage
**Age**			**Tribal affiliation**		
21–39	109	54.8	Cheyenne River	25	12.6
40–49	36	18.1	Crow Creek	12	6.0
50–59	33	16.6	Lower Brule	4	2.0
60–69	11	5.5	Pine Ridge (Oglala)	33	16.6
> 69	10	5.0	Rosebud	31	15.6
**Gender**			Sisseton-Wahpeton	5	2.5
Female	117	58.8	Standing Rock	12	6.0
Male	82	41.2	Yankton	30	15.1
			Unknown	47	23.7
**Total**	**199**		**Total**	**199**	

Tables 2, 3 and 4 present univariate analysis results that show variables significantly associated (*p* < 0.05) with having never had a cancer screening in their life. Overall, 56% of eligible participants (n = 48 for people aged 50 years or older) had ever had a colorectal cancer screening (e.g., fecal occult blood test, colonoscopy, or sigmoidoscopy). More specifically, about 35% of eligible participants had ever had a fecal occult blood test and 36% of participants had ever had a colonoscopy or sigmoidoscopy. About 84% of eligible women (n = 56) aged 40 years or older had ever had a mammogram and 81% of eligible women (n = 117) aged 21 years or older had ever had a Pap test.

Variables that were significantly associated with having never had a colorectal cancer screening are listed in Table 2, including having never heard of colorectal cancer screening, receiving social services, annual household income lower than $25,000, and having to travel long distance (>60 miles one-way) to PCPs.

Variables that were significantly associated with having never had a mammogram are listed in Table 3, including having never heard of mammogram and having to travel long distance (>60 miles one-way) to PCPs. Among those who had never had a mammogram or who hadn't had one in more than three years, top reasons were: never thinking about having it, not having any problems, never been told by doctors to do so, not knowing it is needed, and putting it off.

**Table 2.** Colorectal cancer screening for people aged 50 years or older.

Characteristics	n = 48	% of participants who never had a colorectal cancer screening	*P* value
**Receiving social services**			< 0.05
Yes	17	64.7	
No	31	32.3	
**Annual household income**			< 0.05
≤ $25,000	13	61.5	
> $25,000	33	33.3	
Unknown	2	50.0	
**Having heard of colorectal cancer screening**			< 0.005
Yes	42	35.7	
No	6	100.0	
**Travel distance to PCPs (one-way)**			< 0.05
≤ 60 miles	39	35.9	
> 60 miles	9	77.8	

**Table 3.** Breast cancer screening for females aged 40 years or older.

Characteristics	n = 56	% of participants who never had a mammogram	*P* value
**Having heard of mammogram**			< 0.001
Yes	50	10.0	
No	6	83.3	
**Travel distance to PCPs (one-way)**			< 0.05
≤ 60 miles	46	8.6	
> 60 miles	10	50.0	

Variables that were significantly associated with having never had a Pap test are listed in Table 4, including having never heard of Pap test, never told to get a screening by a doctor, no car, no PCPs, having to travel long distance (>60 miles one-way) to PCPs, unemployed, receiving social services, medical expense payment, self-rated health status, education level, marital status, and age.

Over half of the participants (51.9%) without a car had never had a Pap test, and 40% of participants who had to travel more than 60 miles one-way to PCPs had never had a Pap test. The majority of participants who had never had a Pap test rely on Indian Health Service (IHS) for medical expense payment. Regarding age, the majority of participants who had never had a Pap test were under 40 or over 49. Regarding education, those who had never had a Pap test tended to have education lower than college level. About half of the women (46.7%) whose self-rated health status was “Excellent” had never had a Pap test. Marital status was a significant factor as well. Among 55 women who never got married, 34.5% of them had never had a Pap test.

Among those who had never had a Pap test or who hadn't had one in more than three years, top reasons were: never thinking about having it, not having any problems, never told by doctors to do so, putting it off, not knowing it is needed, and being unpleasant.

**Figure 1.**
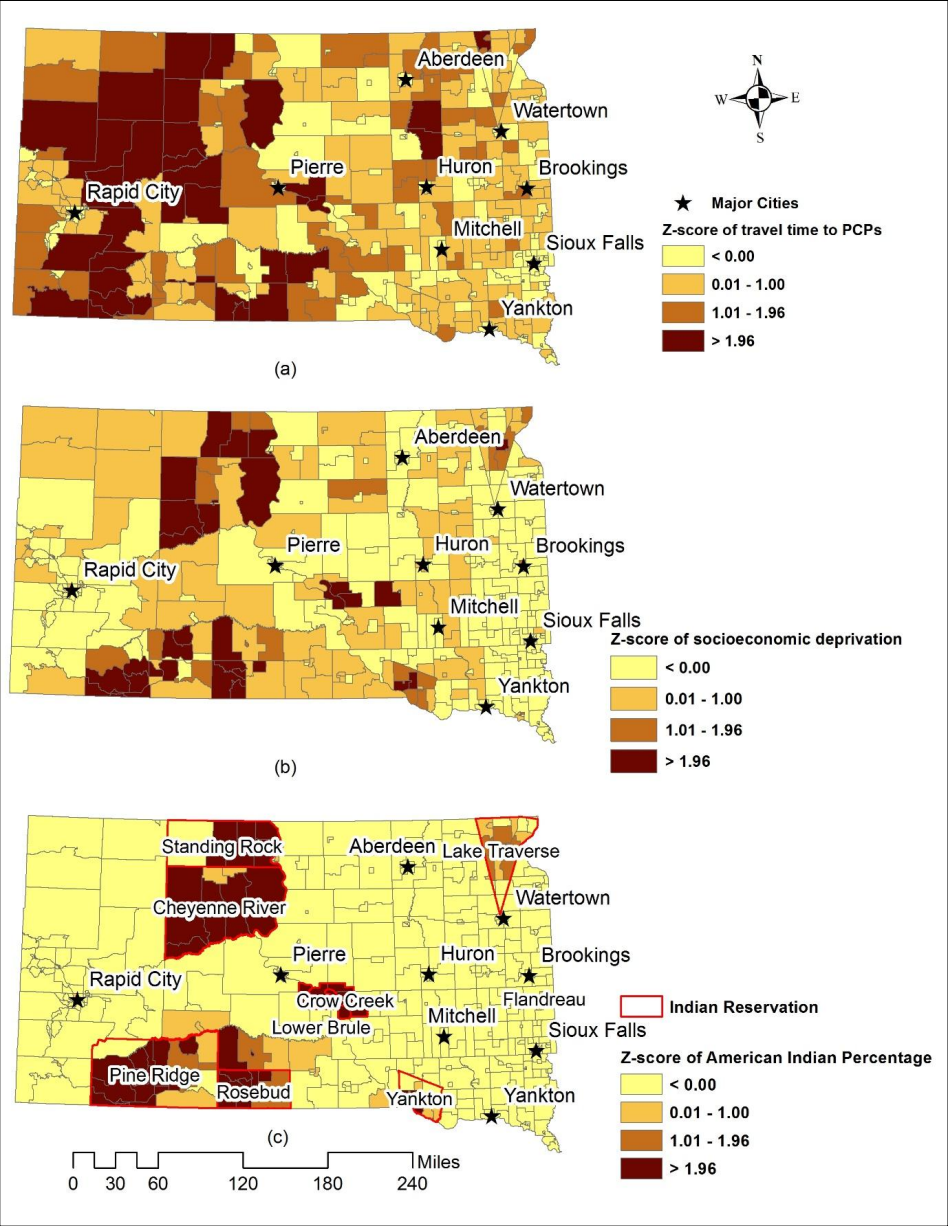
Standardized Z-scores of geographic access to PCPs (a), socioeconomic deprivation (b), and percentage of American Indian population (c).

**Figure 2.**
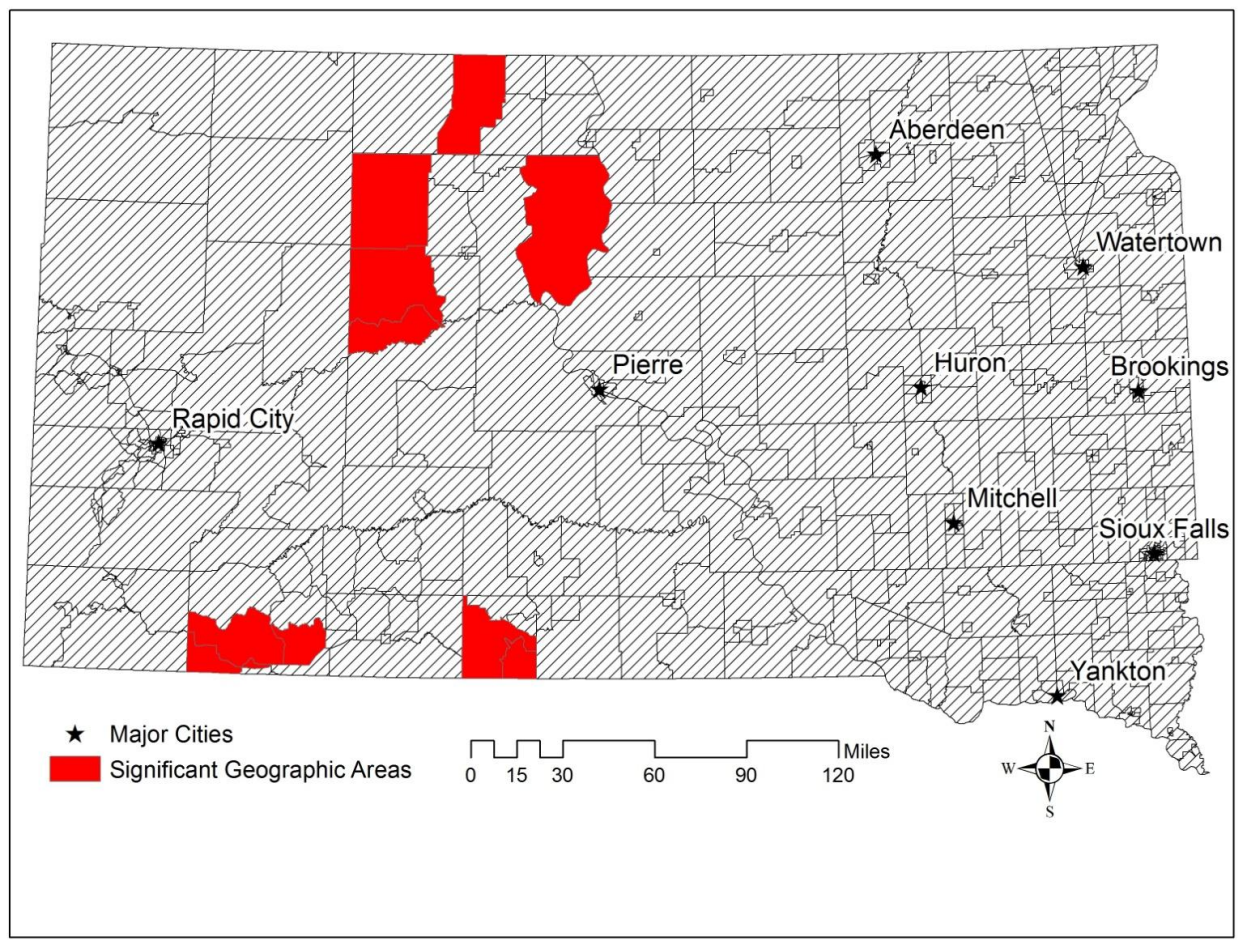
Geographic areas (shown by red census tracts) with significantly low access to cancer screening for American Indian population.

Figure 1 shows Z-scores of geographic access to PCPs, socioeconomic deprivation, and the percentage of American Indian population. We used 1.96 as a cut-off value to identify areas with significantly longer travel time to PCPs, higher socioeconomic deprivation, and higher percentage of American Indian population. Figure 1a suggested that most western South Dakota experienced significantly longer travel time to PCPs compared to the rest of South Dakota. Figure 1b revealed that significantly high socioeconomic deprivation was found in Indian reservations. Figure 2 shows geographic areas of significantly low access to cancer screening for American Indian population (red areas). A total of 10 census tracts were identified, primarily located in four reservations—Standing Rock, Cheyenne River, Pine Ridge, and Rosebud.

**Table 4.** Cervical cancer screening for females aged 21 years or older.

Characteristics	n = 114^a^	% of participants who never had a Pap test	*P* value
**Ever told to get a screening by a doctor**			< 0.001
Yes	39	5.1	
No	66	27.3	
**Having heard of Pap Test**			< 0.001
Yes	102	10.8	
No	12	91.7	
**Having a car**			< 0.05
Yes	87	9.2	
No	27	51.9	
**Having a PCP**			< 0.01
Yes	70	14.3	
No	36	19.4	
**Travel distance to PCPs (one-way)**			< 0.05
≤ 60 miles	99	16.2	
> 60 miles	15	40.0	
**Currently employed**			< 0.005
Yes	65	9.2	
No	47	31.9	
**Receiving social services**			< 0.001
Yes	18	38.9	
No	89	11.2	
**Medical expense payment**			< 0.005
IHS only	39	12.8	
IHS and Medicare	23	52.1	
IHS and other	22	4.8	
Medicare	5	0	
Other	22	13.6	
**Self-rated health status**			< 0.05
Excellent	15	46.7	
Very Good	27	25.9	
Good	46	8.9	
Fair	22	18.2	
Poor	3	0	
**Education**			< 0.005
No High School	6	66.7	
Some High school	14	35.7	
Graduated High School/GED	27	29.6	
Some College	27	11.1	
Graduated from College	23	0	
Advanced Degrees	17	11.8	
**Marital status**			< 0.005
Never married	55	34.5	
Married or living together with a partner	33	3.0	
Separated	7	14.3	
Divorced	15	0	
Widowed	4	25.0	
**Age**			< 0.001
21–39	60	23.3	
40–49	21	0	
> 49	33	24.2	

**^a^** Characteristics with unknown entries are not included in this table. The sum of this column may not equal 114.

## 4. Discussion

This study identified barriers for breast, cervical, and colorectal cancer screening among American Indian population through a community survey in South Dakota. Some common barriers included knowledge about cancer screening, geographic access to PCPs, as well as socioeconomic barriers. Integrating geographic access, socioeconomic deprivation, and the geographic distribution of American Indians, the study identified geographic areas of hard-to-reach populations who had low access to cancer screening in South Dakota.

In this study, we also compared cancer screening rates of our survey sample against those from the Behavioral Risk Factor Surveillance System (BRFSS) in 2014 in South Dakota. Although we conducted the current survey in 2015, we used the BRFSS in 2014 because data in 2015 were not available. We found that 35% of eligible participants had ever had a fecal occult blood test and 36% of participants had ever had a colonoscopy or sigmoidoscopy. BRFSS reported that among American Indians aged 50 years or older, 33% of them had ever had a fecal occult blood test and 47% had ever received a sigmoidoscopy or colonoscopy [17]. We found that 84% of eligible women had ever had a mammogram while BRFSS reported 72% in 2014. For cervical cancer, we found that 81% of eligible participants had ever had a Pap test while BRFSS documented 90% in 2014 [17]. The slightly inconsistent results were likely due to sample bias. We only included 199 participants in this survey sample while BRFSS had 737 American Indian participants. Another possible explanation is different sampling strategies used. The BRFSS used telephone respondents and we conducted face-to-face written surveys.

In this study, having ever heard of cancer screening was found to be significantly associated with cancer screening among American Indians, which is consistent with previous studies. A prior study among American Indians in Navajo Reservation found that participants who had ever heard about colorectal cancer screening were more likely to get screening [18]. Possible reasons for having never heard of cancer screening could be low health literacy or lack of health education. Top reasons listed by participants for having never been screened, such as never thinking about having it, not having any problems, and not knowing it is needed, suggested that lack of health education was a barrier for cancer screening among American Indians in the study. About half of participants in the study only had high school education, which implied health literacy. Although there was only a small portion of participants who had never heard of cancer screening (14% for colorectal cancer, 12% for breast and cervical cancer), over 90% of those had never been screened before. These findings suggested a need for targeted cancer screening education and intervention programs.

This study also found that having never been told by doctors to get a cancer screening was associated with having never been screened before, which suggested that lack of encouragement for cancer screening by health professionals was a significant barrier among American Indians. Similar findings were observed among American Indian population in previous studies [6],[11],[19]. In this study, about 60% of participants responded that they had never been told to get a cancer screening by a doctor or nurse. A prior study also found that American Indians were more likely to experience medical mistrust and dissatisfaction with health care compared with their white counterparts [6]. Findings from this study demonstrated a great need for health professionals to better communicate with American Indian patients the importance of cancer screening. If a doctor recommends cancer screenings to patients, it is highly likely that the acceptance of cancer screening will be increased.

Travel time was a significant factor for all cancer screenings in this study, which corroborated previous studies that distance and transportation were significant barriers for American Indian communities [6],[10],[14],[20]. Because most American Indians resided in rural areas especially western South Dakota who experienced longer travel time to PCPs as shown in Figure 1a, travel distance poses a barrier to seek primary care services. A prior study found that it required longer travel time to access quality primary care because most clinics in rural areas close to American Indian communities were short-staffed [6].

This study revealed that socioeconomic factors also predicted cancer screening rates. Significant socioeconomic variables, including income, education level, employment status, and whether receiving social services, were associated with colorectal or cervical cancer screening in the study. A previous study also found that socioeconomic deprivation was a significant predictor for colorectal cancer late stage diagnosis which was closely associated with colorectal cancer screening [16]. Although all American Indian participants in the study were eligible for Indian Health Service (IHS)-funded health care, the present study identified a significant role of socioeconomic factors in cancer screening, which was consistent with previous studies [12]. A possible explanation might be that current cancer screening intervention efforts were more successful among American Indians of higher socioeconomic status, which suggested a need for targeted intervention programs among people with a lower socioeconomic status.

For cervical cancer, this study identified significant barriers that have been proven significant in previous studies, including marital status, age, self-rated health status, whether or not having a PCP, and socioeconomic factors [11],[15],[20].

This study also identified geographic priority areas (hard-to-reach areas) for a targeted intervention. We found that for participants of lower geographic access (residing in remote rural areas) or lower socioeconomic status, the percentage of having never heard of cancer screening was even higher. This finding demonstrated a great need to develop and implement targeted intervention programs in these identified geographic priority areas.

This research is essential for the development of cost-effective cancer intervention programs. On the one hand, by identifying geographic areas with significantly low access to cancer screening among American Indians, this study could help develop geographically targeted cancer intervention programs through providing crucial information about where hard-to-reach population reside and where to intervene. The geographic areas identified in the study were where American Indians need the most assistance regarding cancer screening. Prioritizing geographic areas that need the most help and distributing resources to those areas could optimize health care resources and ultimately save cost. On the other hand, understanding barriers that were associated with cancer screening can help develop personalized and targeted intervention programs which select recipients of interventions based on these barriers. For example, since this study found that knowledge and socioeconomic factors were significant barriers, health education and intervention materials should be delivered to communities which are characterized by low socioeconomic status or health illiteracy.

Several limitations need to be considered in this study. First, the small sample size in the study might introduce bias in our findings. We recruited participants through American Indian community events which was not a random selection process. Nevertheless, participants in the study did not differ significantly from the general American Indian population in the distribution of age and sex. Second, we used self-report written questionnaires in the study, which might suffer validity problems. Additionally, most of the questions in the survey were closed questions, which might have limited the collection of more in-depth insights from participants regarding cancer screening barriers. However, findings from this study were consistent with previous studies in the literature [21]. Third, Chi-square test as a descriptive test was used to describe the strength of the relationship between participants' barriers and cancer screening. Sophisticated approaches (e.g., multivariate analysis) which could control for confounding variables were not conducted due to the use of categorical variables as well as the small sample size in the study for each cancer screening outcome. Fitting too few samples (data points) with too many parameters (categorical variables) would result in an extremely small error variance. Last, results from this study might not be extended to other geographic areas because of the cultural diversity among American Indians. The purpose of the present study was to understand barriers of American Indian populations in South Dakota (Sioux) and to identify geographic areas for targeted cancer screening intervention. Therefore, extra precaution should be taken when applying the study to other states.

## 5. Conclusion

This study addressed an important challenge of cancer screening among the hard-to-reach population in the United States. We reach out to the American Indian population that has been labeled “hard-to-reach” group and identified barriers for breast, cervical, and colorectal cancer screening among this group through a community survey in South Dakota – a state with 10% of American Indians. The study found that knowledge about cancer screening, geographic access to PCPs (e.g., distance and transportation), as well as socioeconomic factors were significant barriers for breast, cervical, and colorectal cancer screening. Based on these findings, the study integrated geographic access and socioeconomic factors to identify geographic areas where people had low access to cancer screening in South Dakota. Results from the study are important for the development of cost-effective cancer intervention programs.
